# Modelling Patient Behaviour Using IoT Sensor Data: a Case Study to Evaluate Techniques for Modelling Domestic Behaviour in Recovery from Total Hip Replacement Surgery

**DOI:** 10.1007/s41666-020-00072-6

**Published:** 2020-05-03

**Authors:** Michael Holmes, Miquel Perello Nieto, Hao Song, Emma Tonkin, Sabrina Grant, Peter Flach

**Affiliations:** 1 SPHERE IRC, University of Bristol, Bristol, UK; 2 Musculoskeletal Research Unit, University of Bristol, Bristol, UK

**Keywords:** Internet of things, Wearable sensors, Indoor localisation, Sleep, Mobility, Hip replacement surgery, Actigraphy

## Abstract

The UK health service sees around 160,000 total hip or knee replacements every year and this number is expected to rise with an ageing population. Expectations of surgical outcomes are changing alongside demographic trends, whilst aftercare may be fractured as a result of resource limitations. Conventional assessments of health outcomes must evolve to keep up with these changing trends. Health outcomes may be assessed largely by self-report using Patient Reported Outcome Measures (PROMs), such as the Oxford Hip or Oxford Knee Score, in the months up to and following surgery. Though widely used, many PROMs have methodological limitations and there is debate about how to interpret results and definitions of clinically meaningful change. With the development of a home-monitoring system, there is opportunity to characterise the relationship between PROMs and behaviour in a natural setting and to develop methods of passive monitoring of outcome and recovery after surgery. In this paper, we discuss the motivation and technology used in long-term continuous observation of movement, sleep and domestic routine for healthcare applications, such as the HEmiSPHERE project for hip and knee replacement patients. In this case study, we evaluate trends evident in data of two patients, collected over a 3-month observation period post-surgery, by comparison with scores from PROMs for sleep and movement quality, and by comparison with a third control home. We find that accelerometer and indoor localisation data correctly highlight long-term trends in sleep and movement quality and can be used to predict sleep and wake times and measure sleep and wake routine variance over time, whilst indoor localisation provides context for the domestic routine and mobility of the patient. Finally, we discuss a visual method of sharing findings with healthcare professionals.

## Introduction

The UK health service sees around 160,000 primary total hip and knee joint replacements performed every year within the National Health Service [18]. This number is expected to increase with a growing more active population in the UK [21].

Surgical intervention is, however, only part of a patient’s journey. After a hip or knee replacement, up to 30% of patients experience long-term pain after surgery [4]. In a European collaborative study of 1327 patients with total hip replacement, results suggest between 14 and 36% of patients did not improve symptoms or were worse 12 months after surgery [15].

Poor outcomes include continuing pain and functional problems and a longer term impact on increased healthcare utilisation [4]. With changing expectations of surgical outcome and demographic trends [19], conventional assessments of health outcomes must evolve to keep up with these changing trends.

After surgery, patients routinely receive a follow-up appointment which can frequently be carried out by a different surgeon or registrar, creating a fractured experience of aftercare for the patient. Various strategies have been proposed to increase efficiency whilst maintaining quality and patient acceptability, such as the use of ‘virtual clinics’ [34]. Other than the consultation itself, assessment of health outcomes generally relies on self-reported outcome measures such as the Oxford Hip Score [9]. These can assess various health outcomes including pain, function, and aspects of quality of life but can be limited by self-report, with many lacking a theoretical basis [27, 29].

Previously, research has explored the relationship between PROMs and objective measures, notably performance-based tests such as timed walks or sit-to-stand tests [6]. Such objective measures are administered in controlled, laboratory style settings and may not reflect levels of activity in daily life. Multimodal sensor systems present in the domestic settings, such as those used in ambient-assisted living scenarios [24], allow assessment of behaviour and activity in a natural setting.

Establishing a relationship between PROMS and multimodal sensor data permits us to develop effective methods of passive monitoring and recovery after surgery. Providing a further data source, alongside PROMS, may allow for relatively timely intervention in the event of complications and thus potentially improving patient outcomes.

In this paper, we introduce two cases from the HEmiSPHERE study [12], the first clinical application of the SPHERE IoT sensor network [10, 36]. This paper presents an initial analysis of long-term observational data from three participant homes (Section 4) to evaluate whether IoT sensor data can be used to produce informative trends of patient behaviour during recovery from total hip replacement surgery, using statistical analysis and machine learning techniques. Long-term trends in movement, location and posture activity are visualised to show distinct patterns of patient domestic behaviour (Fig. 10). Finally, generated classifications are compared with PROMs for validation of trends evident in predictions (Section 5). We show that trends in domestic behavioural data can be reliably and accurately generated using the SPHERE sensor network and that these trends are indicative of patient recovery from hip replacement surgery.

In this study, data from three participants are presented for comparison between patients in recovery from surgery and for evaluation in reference to Patient Reported Outcome Measures. Participant A is used as a control, having not undergone surgery. Both Patients B and C are in recovery from total Hip replacement surgery.

## Related Work

Key indicators relevant to PROMS include movement patterns (such as room to room transfers), patterns of improvement (establishment or divergence from routine), high-level activities undertaken (such as cooking or cleaning) and sleep (e.g. hours sleeping, quality of sleep). This study focuses on sleep, movement and domestic routine by analysing and classifying three attributes of patient behaviour—indoor location, movement and activity class. In this section of the paper, the authors briefly introduce literature on the methods selected to perform the analysis.

### SPHERE: a Sensor Platform for HealthCare in a Residential Environment

SPHERE is an interdisciplinary research project which aims to develop sensor technologies capable of supporting a variety of practical use cases, including healthcare- and ambient-assisted living outcomes. An additional goal of SPHERE is to build systems that are considered acceptable by the public and which are flexible and powerful enough to function well in a broad variety of domestic environments [35, 36].

**Fig. 1:**
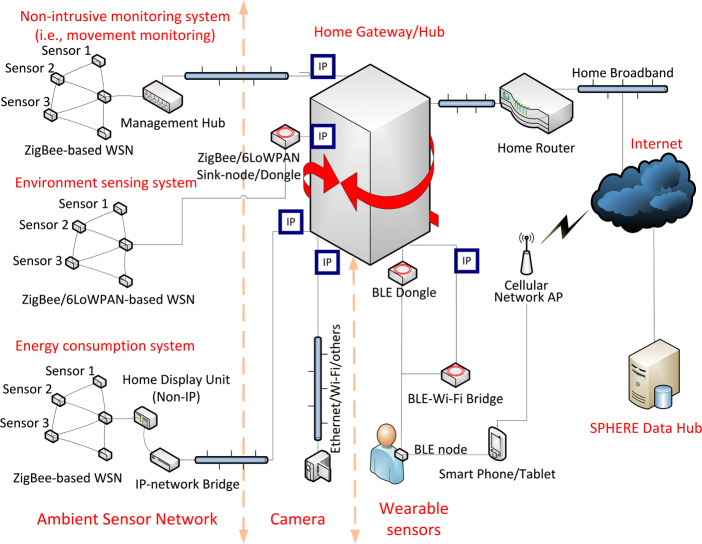
The SPHERE sensor platform consists of multiple subnetworks of sensors including environmental sensors, smart utility meters, cameras and wearable sensors. Sensors in the network communicate using Bluetooth Low-Energy (BLE) gateways

‘Smart home’ systems development has primarily taken place in laboratory settings [1], or, as in the SPHERE project, in a customised home [30]. In 2017, the SPHERE project began to deploy a multimodal sensor network (Fig. 1) into dozens of homes in the South West of England. In 2018, the HEmiSphere project began deploying the SPHERE sensor network in the homes of patients as they underwent total hip or knee replacement surgery.

As shown in Fig. 1, the SPHERE sensor network provides an overlapping mesh of sensors within a domestic environment. The network incorporates a number of sensor subsystems, including video systems, environmental sensors, electricity and water meters and wearable sensors communicating over Bluetooth Low-Energy (BLE) connections. In this research, the authors focus on RSSI and accelerometer data collected from the wearable subsystem.

### Indoor Localisation

Indoor localisation [23, 33] is an important area of research for behavioural analysis in residential healthcare. The ability to predict the location of a patient not only gives insight into domestic routine and habitation but allows other information to be physically contextualised.

The SPHERE network provides a mesh of Received Signal Strength Indicator (RSSI) fields. As in literature [23, 33], RSSI has been used to *fingerprint* locations within a space by learning the discriminant RSSI vectors from a moving average [23].

Initial testing of RSSI fingerprinting within the SPHERE sensor network [13], using a multilayer perceptron network for location classification, yielded positive results. On a single sample home, the network achieved above 80% classification accuracy on a limited set of indoor locations.

### Measuring Movement with Wearable Accelerometers

Accelerometers are sensors that measure the rate of change in velocity and can be used to measure movement of a person [22, 37]. The accelerometers used in the SPHERE sensor network (Fig. 1) are tri-axial, meaning they record acceleration in three dimensions, *x*, *y* and *z*. In [22, 37], wrist-worn accelerometers are used to monitor acceleration magnitude (Equation 1), which is the square root of the acceleration vector. The magnitude gives a single signal which describes the magnitude of acceleration regardless of the axis, or direction, of acceleration. Magnitude is useful in modelling the force of movement, when specific orientation information is not necessary.

1 $$  A = \sqrt[]{x^{2} + y^{2} + z^{2}} $$

The wrist-worn sensor will show spikes in magnitude indicative of movement such as ambulation (e.g. walking or running), hand or arm movements (e.g. chopping vegetables) or posture change (e.g. rolling over in bed). In aggregate, movement data can also be used to study activity levels over an extended period of time, such as sleep quality and rhythm [2], using for example sleep quality and consistency measures proposed and used in actigraphy.

### Classifying Posture and Ambulation Activity

Activity recognition using wearable and mobile devices has been a major focus for the recent years [3, 14, 17, 25, 28]. From a device prospective, mobile phones, smart watches and wrist bands have the dominant source of data, which normally captures the acceleration signal around the body of the users. In this paper, we also focus on the 3-axis acceleration data obtained from a wrist band, which is one of the standard approaches used in the field.

## Case Study

The case study presented in this paper develops the analysis presented in prior work by the authors [13]. The methods selected for analysing location, movement and activity have been piloted in prior work using a single simple home over a short time period. In this work, we present a study of three participant homes—two with patients in recovery from total hip replacement surgery—over a 3-month time period. The three participants are aged between 50 and 85 years of age. Whilst the homes of each participant have different rooms and layouts, the commonalities such as bedroom, kitchen, bathroom and living room may be informative in terms of Activities of Daily Living such as cooking, bathing, sleeping and leisure.

The ambition of this case study is to show, for the first time, that analysis of data collected using the SPHERE sensor network (see Section 2.1) can produce outcomes indicative of trends reported in their patient reported outcome measures for mobility, sleep and routine. In this section of the paper, we describe the data collected, ethical considerations and analytical methods used to learn and recognise behavioural patterns expressed within the data.

### Data Collection

Three case study homes have been selected from the cohort of participants. Each selected case study home has one participant wearing a SPHERE wearable device.

Participant home A is selected from the overall SPHERE cohort. The home is used as a control, where the participant is not in recovery from surgery. Participant home A allows us to visualise a baseline routine of domestic activity. Participants in homes B and C both underwent total hip replacement surgery within the first 2 weeks of observation. Participants in homes B and C were observed for 3 months following surgery.

Participants in homes B and C rated their health outcomes over the recovery period. Self-reported PROMS include a sleep quality survey [8] and the Oxford Hip Score. [9] PROM data has been collected for participants in homes B and C for comparison with analysis made from data collected using the SPHERE Sensor network.

The SPHERE system has been installed in the residence for 5 months. In this paper, we focus on analysis of the first week of installation, so as to give an overview of the methods used for analysis and visualisation of data. Figure 1 shows the physical architecture of the SPHERE platform, of which one subsystem is the wrist-worn Bluetooth Low-Energy (BLE) wearable device. The wrist wearable harbours a tri-axial accelerometer and broadcasts over Bluetooth at 25Hz.

### Ethics: Data Collection and Publication

The data used in this study has been collected as part of the SPHERE [35, 36] and HEmiSphere [12] projects. Ethical approval for HEmiSPHERE was granted on 22/06/2017, 17/SW/0121. The participants in this case study have provided consent for data to be recorded within their home. Participation in both the SPHERE and HEmiSPHERE projects is voluntary, and participants are at liberty to exit the experiment at any time.

Due to the sensitive nature of data collected within a real-world residential environment, data used in this study is not being made public alongside this paper. A data set of activity and location annotated SPHERE sensor data, recorded during short scripted experiments in the SPHERE House *(The SPHERE Challenge)* [31], is available online.

### Methods

In this section, we present an overview of methods used to generate the three classification metrics: in-door localisation, movement and activity classification.

#### Classifying Location Using RSSI Fingerprints

**Fig. 2:**
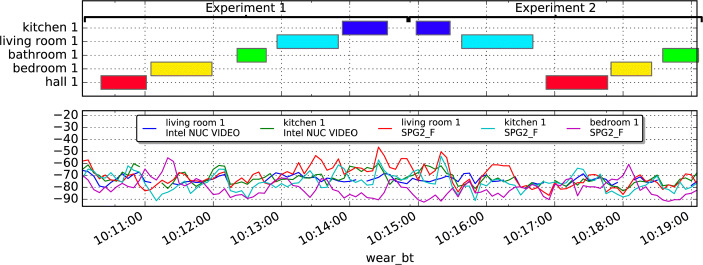
Visualisation of technician walk around with annotated locations (top) and RSSI data (bottom) for house A. The technician performed a set of two annotation experiments, going through every room in the house. The bottom subplot shows the RSSI intensity from the wrist-band to 5 devices

To develop a localisation training set for the home, during installation of the SPHERE sensor network, a technician performs an annotation procedure called a ‘technician walk-around’. The technician carries the wearable device to each room in the home, annotating the start and end times in each labelled location. The technician walk-around was repeated prior to the sensor network being removed from the home. Figure 2 visualises the technician walk around, showing annotated location labels above a plot of the RSSI signals for the corresponding period.

RSSI levels between the wrist-worn wearable and each installed receiver within the home have been recorded. Using a 1-s window, a vector of RSSI values is constructed to represent the position of the participant. For each gateway, the sum, mean, minimum, maximum and variance in RSSI are calculated across the second. If the wearable is out of range of all gateways, no data is produced. If the wearable is out of range of one or more, but not all, gateways then values for those gateways are substituted with the constant *rssi* = − 100*db*. This value was chosen as it is minimally beyond the maximum *dB* threshold for connection.

A multilayer perceptron artificial neural network (MLP) with three hidden layers, 10 nodes per hidden layer, was trained to classify the location of the wearable based on RSSI vectors. To train the classifier, the annotations taken during the technician walk-around activity (Fig. 2) were used to label the training and test set of vectors.

In initial testing, training on data from the first technician walk-around and testing on data from the second provided poor results in some important locations, such as the bedroom in home A. This may be, in part, due to changes made within the home during the observation period, such as movement or addition of furniture, which change the RSSI signals. For this initial study, we train the classifier using a random sample of data from both walk-arounds.

The set of labelled vectors were shuffled and split 60/40 between training and testing sets. Parameters including momentum, learning rate and solver were tuned using grid search, with the optimal configuration selected based on training set performance ascertained using Sci-kit Learn’s MLPClassifier ‘score function’. A separate classifier was tuned and trained for each participant data set. Classifiers were then tested on an out-of-sample test set. The best performing solver was the ‘Adam’ [16] algorithm. Results of training and testing are presented in Section 4.1.

#### Classifying Movement and Sleep-Wake Routine

Movement is calculated by the magnitude of acceleration (1), as given by the mean tri-axial accelerometer readings from the wrist-worn wearable, over a 1-min window. This approach has been successfully demonstrated in [37]. The wearable device transmits acceleration in *x*, *y* and *z* dimensions at 25 Hz. Acceleration magnitude was calculated for each 1-min of accelerometer data. For each 1-s window, the mean, min, max and standard deviation of magnitude were calculated. Using the nparACT Actigraphy package [5], the movement data is used to estimate a routine of the participant including most and least active periods, and sleep-wake cycle. Results of training and testing are presented in Section 4.2.

#### Classifying Posture Activities Using Tri-axial Acceleration

**Fig. 3:**
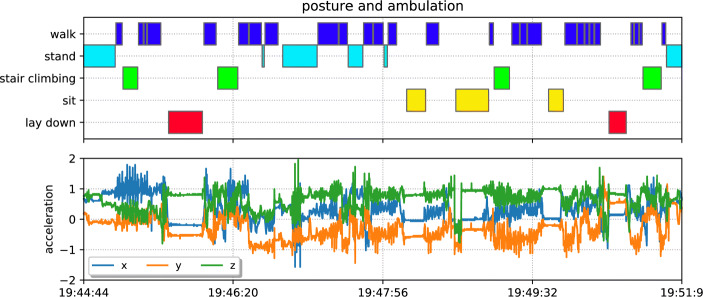
Visualisation of day in fast-forward experiment with the annotated activities (top) and the accelerometer values (bottom) for house B. The activities are manually annotated by inspection of a video recorded from a head-cam camera

We now describe how to obtain the inference over certain activities using the acceleration signal from the wearable device. To train the classifier, we collect a set of annotated datasets via performing scripted experiments in different homes. These scripted experiments are designed as follows. We ask the participant to simulate their daily life (e.g. wake from bed, go to the kitchen, stay in the living room, back to the bedroom) whilst wearing a head-mounted camera (Fig. 3). The whole process normally takes from 5 to 10 min, after which the recorded videos are annotated with five ambulation and postures: (1) lay down, (2) sit, (3) stair, (4) stand, (5) walk. Including the three participant houses above, we use a total number of 10 scripted experiments from different houses to train the activity classifier. Given the amount of available training data, we selected to adopt existing feature engineering approaches to process the wearable acceleration data. The features we considered in this paper are calculated according to the common sliding window approach [32]. We apply a sliding window of the length of 6 s, with a step size of 3 s. Within each window, we first generate a gravity-free signal by computing the L2 norm of the 3-axis acceleration signal, then we calculate the mean and standard deviation from this norm together with the existing acceleration from 3 axes. Additionally, for each window, we also calculate a normalised histogram over the gravity-free signal (with 10 bins from − 4 to 4).

We split the generated features and the annotated activities using stratified 5-fold cross-validation. We train a random forest with the default parameters from the Python library Scikit-learn; 100 decision trees, Gini index as a split criterion, a minimum of 2 samples per split and at least one sample per leaf, the number of features of each tree is fixed to square root of the total number of features, and the number of samples for the bootstrap is always the same as the original number of samples but randomly selected with replacement. We test the training performance of random forests with several random seeds and choose the one with the highest training accuracy. The out-of-sample test performance is then estimated from the validation folds.

## Results

In this section, we present results for training of indoor localisation and activity classifiers using the methods described in Section 3.3 along side 3-month observational data and classifications for location, movement and posture activity.

### Indoor Localisation

Table 1 show the test set performance of the trained MLP indoor localisation classifier (see Section 3.3.1) for each participant home, based on the technician walk-around labels, as described in methodology Section 3.1.

**Table 1:** Location classifier test-set results for participants

Participant	Accuracy (%)	Precision	Recall	f1-score	Support
A	81	0.81	0.81	0.81	163
B	74	0.76	0.74	0.75	897
C	71	0.73	0.71	0.72	464

**Fig. 4:**
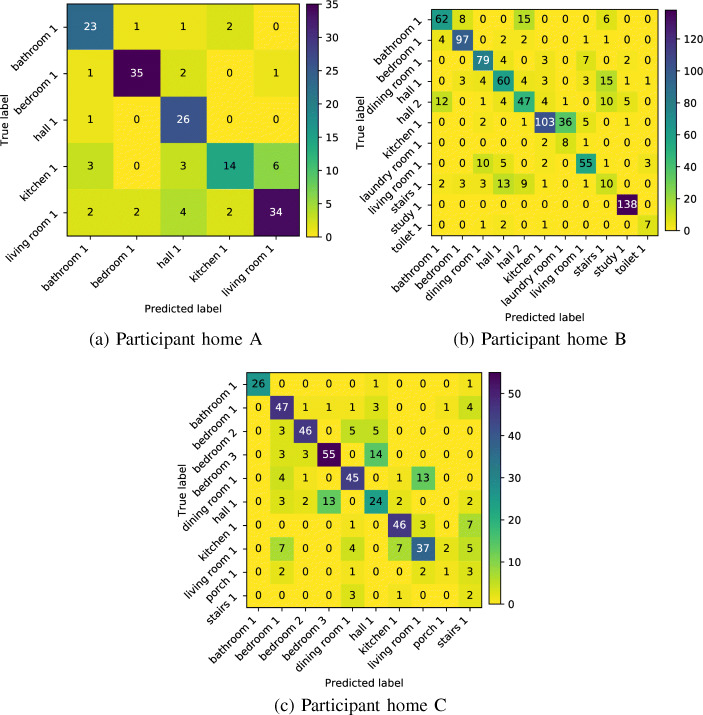
Confusion matrices for localisation classifiers for each house. Each confusion matrix shows high values in the diagonal, denoting a strong true positive counts. In home B, the halls 1 and 2 are commonly confused by the stairs 1, and the kitchen 1 and the laundry room 1 seem difficult to differentiate as well. In home C, the bedroom 3 is confused with the hall 1, whilst the porch 1 and stars 1 are occasionally predicted when the true location was living room 1, kitchen 1, dinning room1 or bedroom 1

The classifier test results show that for all three participant homes, the indoor localisation model proposed achieved test-set accuracy between 74 and 81%. Participant home B had the largest number of locations, with 11 annotated locations, and achieved the lowest overall accuracy. Figure 4 show the confusion matrices for each localisation model. The confusion matrices show the counts for each type of prediction and highlight where models perform well or poorly. The confusion matrix B shows that the classifier performed poorly in distinguishing between the kitchen and the adjoining laundry room, and the hallways and the stair wells.

**Fig. 5:**
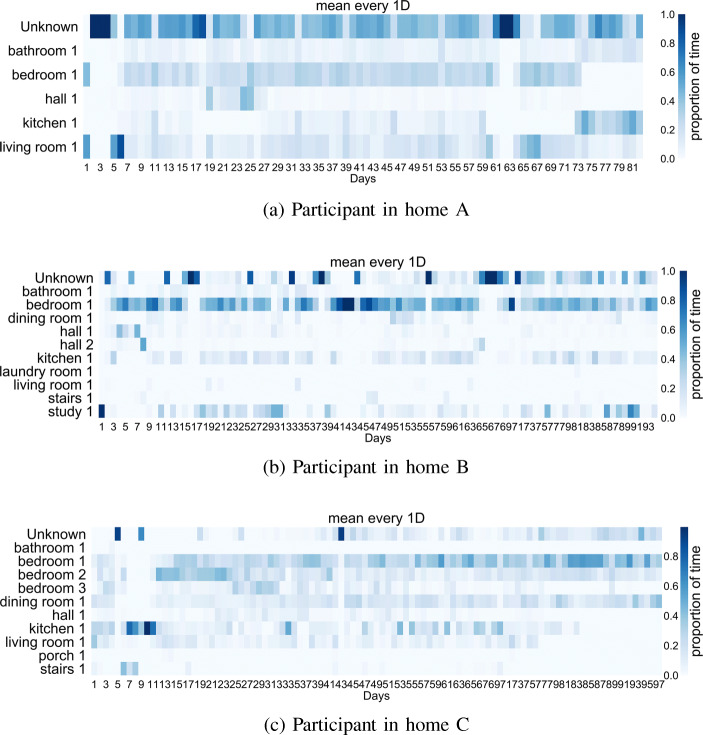
Location classifications: percentage time in location per day. The control home A **a** shows long time spent in an “Unknown” location meaning that the wearable was out of reach and denoting time spent outside of home. Participant in home B **b** shows occasional time spent outside of home increasing over time, whilst participant in home C **c** shows an increasing amount of time spent in bedroom 1

The trained classifiers have been applied to the 3-month observation period for each participant home. Figure 5 shows the distribution of time spent, by each participant, in each location of their home. The ‘unknown’ location label is added here to denote time when the participant is not in range of the sensor network, or the sensor network is turned off. Other data confirms that the sensor network is on during the observed periods allowing ‘unknown’ to represent time spent outside of the home.

**Fig. 6:**
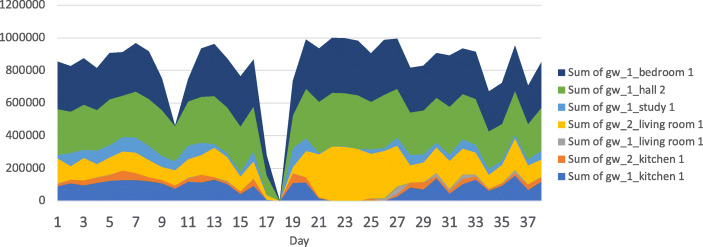
Counts of Bluetooth low-energy gateway sensor messages for home B over 37 days

The localisation classification results (Fig. 5) for the observed 3-month post-operative recovery period highlight some interesting differences between our healthy control home, A, and patients in homes B and C. The participant in home A (Fig. 5a), our control participant, regularly spends time in each location of the home but occupies an ‘unknown’ location, here representing time outside of the home, for some period on each week day; less time is spent outside of the home on weekends. The participant in home B (Fig. 5b), a post-operative patient, increases the frequency of their time away from home in the final month of observed behaviour. The participant in home B decreases their time in the bedroom over the same period and slightly increases time spent in the kitchen and study locations of the home. Dis-similarly, the participant in home C (Fig. 5c), again a post-operative patient, shows an increase in time spent in the bedroom across the observed recovery period and their time spent in the kitchen and living room decreases. These patterns of domestic behaviour indicate potentially different outcomes for recovery from surgery, where location data for the participant in home B shows an increase in mobility and activity outside of the home, whilst for C, the range of locations in use decreases over time. However, the longer term predictions do highlight intermittent problems. The error in location predictions for home B, by which the participant appears to inhabit the bedroom for several days (see Fig. 5b), is shown to be caused by several gateways failing to report data. Figure 6 shows a stacked area chart of messages sent through each Bluetooth low-energy gateway in the home. Between days 21 and 25, three sensors are absent from the dataset.

This error highlights a weakness in RSSI fingerprinting with supervised learning. RSSI fingerprints learned during training cannot be representative of the network fingerprints during periods where one or more gateways are temporarily offline, either due to fault or disconnection. Whilst these problems are unlikely to occur in a laboratory setting, in the wild such conditions may occur due to hardware or power failure. The erroneous location classifications will continue until the network is returned to a fully operation state. One solution to this would be to train with synthetic data, incorporating synthetic failures, or to train with an ensemble of binary classifiers. Future work to improve methods will incorporate research in to fault tolerant classifiers.

### Movement and Routine

**Fig. 7:**
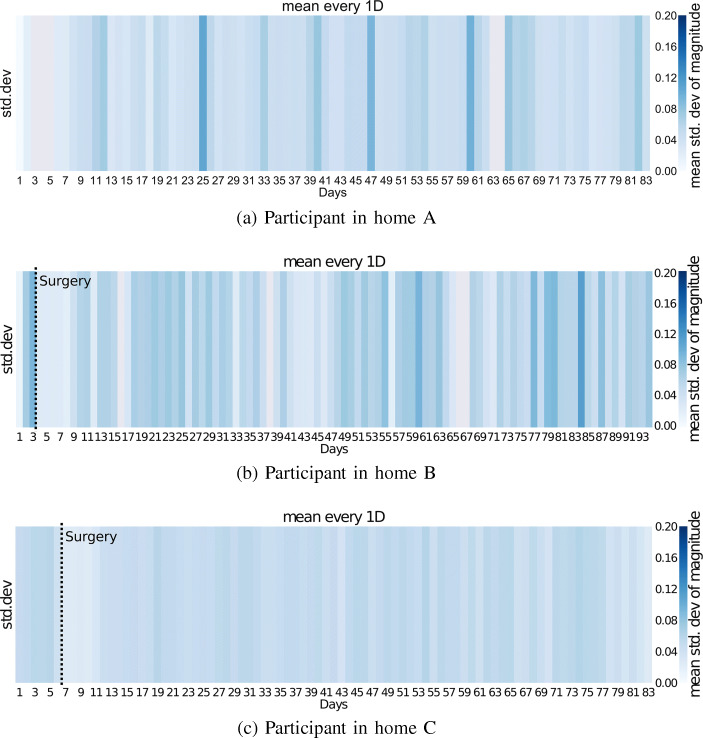
Mean standard deviations in accelerometer magnitude per day

Raw accelerometer data is used to produce magnitude statistics, using the method described in Section 3.3. Figure 7 shows the mean standard deviation of magnitude per day, across the observed period. The standard deviations in acclerometer magnitude show how varied, per minute, acceleration magnitude is on average across each day. The higher the average standard deviation, the more forceful acceleration has occurred.

Movement data extracted from the wearable accelerometer (Fig. 7) shows a stark difference in movement behaviour between patients B (Fig. 7b) and C (Fig. 7c). Whilst the patient in home B shows a healthy pattern of movement and rest, similar to our control in home A (Fig. 7a), the patient in home C (Fig. 7c) shows very low levels of movement throughout the post-operative recovery period. Whilst the patient in home B increases their movement across the period, the patient in home C decreases their overall levels of movement.

Accelerometer data may be used as a data source for actigraphy, a family of approaches used to support the study of sleep and circadian rhythms [2].

As the SPHERE wearable reports data only when the participant is in the home, a comprehensive dataset is not available. Consequentially, it is necessary to impute missing values before these methods can be applied. Using the simplest method, we may impute missing values to zero. We have also explored the impact of using alternative methods, such as the use of time-series analysis to interpolate missing values in a manner that takes into account the nature of the dataset, although a detailed discussion of these is beyond the scope of this paper.

We use version 0.8 of the nparACT package to analyse the data. This is a recently published tool for non-parametric analysis of actigraphy data [5], able to calculate start times and average activity values of the most active 10 h of the day (M10) and of the least active 5 h of the day (L5). Additionally, interday stability and intraday variability are made available. These values can be compared against the sleep PROMs for the purposes of data validation. Intraday variability in particular is used as a marker for sleep-wake cycle disturbances, such as night-time activity and sleep during the daytime [11], and is therefore relevant to the study of conditions or interventions that imply disturbed sleep. We expect that patients recovering poorly may suffer more post-surgery pain or discomfort resulting in poor reported sleep.

L5 predictions and participant predicted start of sleep times are presented in Table 2. It is worth noting that the time difference between the start of L5 and participant predicted start of sleep time is, across the whole time period, reasonably small for most participants. Whilst participant A has a well-established sleep routine, their case presents some difficulty because the participant is away from the home for extended periods, as well because the participant’s weekend routine, as can be visually established by reviewing the relevant graphs (Fig. 10), differs significantly from their weekday routine. As they are more likely to be at home during this time, the dataset as a whole is likely to be biased towards their weekend habits.

**Table 2:** Variation in measures of circadian rhythm, including *ΔT* - difference between start of L5 (lowest 5 h) and participant predicted start of sleep time

	A	B	C
*ΔT* offset (onset of L5-participant prediction)	+ 120m	+ 60m	− 35m
IV during first 5 days	0.23	0.2	0.64
IV prior to follow-up 2 period	NA	0.9	1.05
IV during final 6 weeks	NA	0.87	1.15
Reduction in RA post-op	NA	− 0.7	− 0.9
Intraday variability across 3 months	0.23	0.2	1
RA (Relative amplitude) across 3m	0.59	0.3	0.14

Intradaily variability (IV) is used to establish how regularly an individual switches between rest and activity. From visual inspection of Fig. 10, in particular the accelerometer data, we note that participant C is almost always somewhat active, although there is a period of approximately 5 h per night in which they are somewhat less so. Additionally, they are seldom very active and there are signs of periods of rest during the day. This suggests that IV is likely to be high for this participant. The relative amplitude (RA) measure is reduced following the operation for both B and C, implying that the participant’s circadian rhythm is disrupted. By comparison, participant B eventually regains a pattern of low activity during the night, suggesting that their final IV score should be reasonably low. A, similarly, has a clear pattern of rest, with some slight variance.

Comparing this with PROMS, these findings coincide with participant B’s mention of broken sleep, with slightly better gradual improvement than participant C. Additionally, the evidence supports the conclusion that participant C’s sleep does not improve significantly by the end of the study.

### Activity Classification

**Fig. 8:**
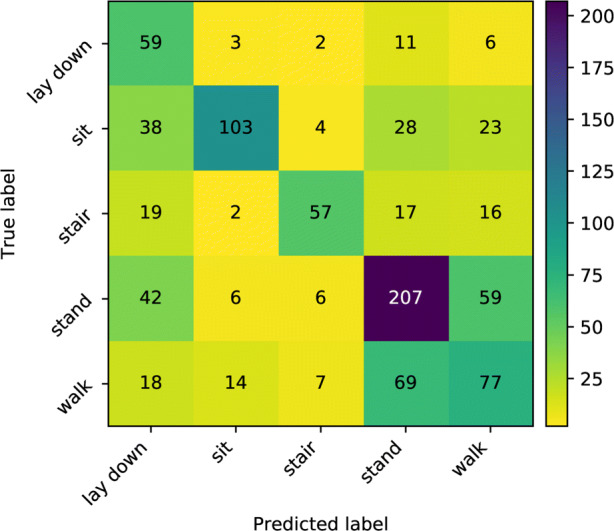
The confusion matrix on activities obtained by 5-fold cross-validation. Shows a strong component on the diagonal denoting correct predictions, whilst the activity stand is sometimes predicted as walk

**Fig. 9:**
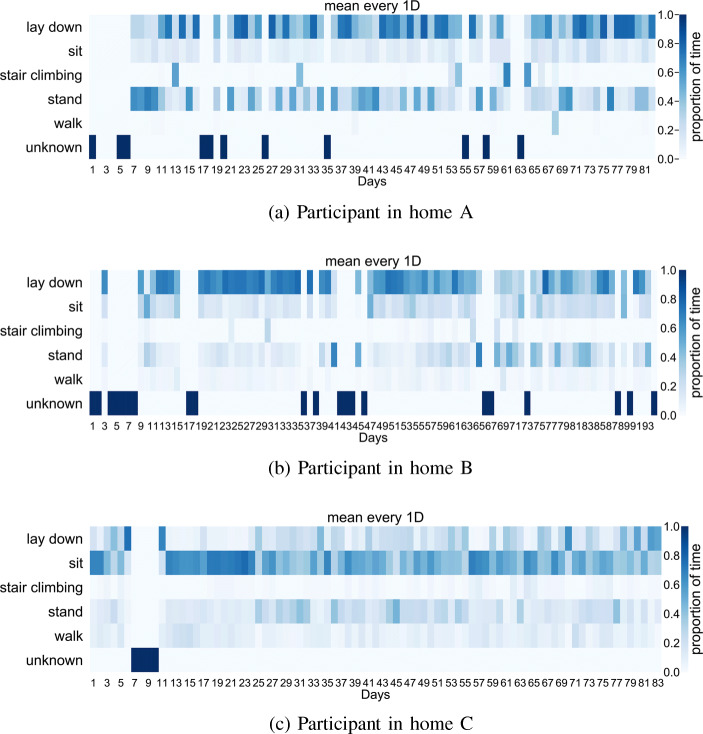
Activity classifications: percentage time in posture-activity per day

The 5-fold cross-validation confusion matrix is given in Fig. 8. As indicated by the confusion matrix, the classifier is able to distinguish most of the ambulation and postures. One exception is that the activity walk is very likely to be mis-classified as stand. Whilst quantitatively this shows a slower performance compared with other activities, it should be aware that walk and stand are often exchanged rapidly in real life, which creates a higher difficulty to distinguish them. On the other hand, given the main purpose of the activity classifier is to evaluate the recovery progress of the patient by checking the stationary and non-stationary activities, the classifier is hence showing reasonable performance here. Figure 9 further shows the inferences over the whole period for each of the participant, which captures the proportion of time spent on each ambulation and posture.

Regrading the inferred activities, as shown by Fig. 9, the most significant difference among the participants can be found on the time spent with lay down and sit. Whilst for participants A and B most time are spent on lying down, participant C is inferred as spending most time on sitting. Since in the results above we have demonstrated that the classifier is mostly correct on classifying sit and lay down, here we can initially exclude the possibility that the classifier is wrong and shows different results among the three participants. One possible explanation here is that participant C did spend extra time sitting on the bed due to the poor recovery indicated by the PROM information. Similarly, if we compare participants A and B, we can see there is not a clear pattern on the lay down time for participant A whilst a clear reduction of time is shown for participant B. Given that the PROM information from participant B indicates a good recovery, these results also demonstrate the effectiveness of using the SPHERE system to monitor the recovery progress.

## Comparison with PROMS

Descriptive scores on one of the PROMs within HEmiSPHERE as shown in Table 3 demonstrate that participants B and C show improvement over time in pain and function, measured by the Oxford Hip Score (OHS) and the Hip disability and Osteoarthritis Outcome Quality of Life (HOOS QOL) subscale.

**Table 3:** Descriptive scores on one of the PROMs scores within HEmiSPHERE

Measure	Participant	BL	FU1	FU2	FU3
Oxford Hip	B	18	23	35	42
Score*	C	32	33	47	47
HOOS QOL	B	37.50%	37.50%	62.50%	100%
subscale**	C	68.75%	62.50%	75.00%	75%

*The Oxford Hip Score is a value in the range [0 − 49], where 0 is the most severe pain and 48 indicates the least symptoms. **The Hip disability and Osteoarthritis Outcome Score Quality of Live (HOOS QOL) subscale is a normalised scale in percentage where 100% denotes no problems, and 0% denotes extreme problems [20]. *BL*, baseline—pre-operative; *FU1*, follow-up one—4–9 days post-operative; *FU2*, follow-up two—6 weeks post-operative; *FU3*, follow-up three—12 weeks post-operative

Participant B demonstrates a slightly better gradual improvement in pain and function between baseline (pre-surgery) and follow-up 1 (within 2 weeks following surgery) than participant C which remains the same, i.e., 32/33. Due to the limitations in how much we can infer from single participant scores within the PROMs, these findings have been triangulated with qualitative interviews conducted with the patients in HEmiSPHERE.

Interviews were conducted by an experienced qualitative researcher with all patients before surgery and approximately 2 weeks after surgery [26]. In-depth interviews using probes and prompts provides understanding of lived experiences.

Interviews were transcribed, anonymised and imported into the qualitative data management software QSR International’s NVivo 11. Interviews lasted between 45 and 60 min. A series of open-ended questions followed a topic guide.

- **Pre-surgery**
  - Route to referral for surgery
  - People living in the household
  - Previous experience of health technology (home, wearable, apps)
  - Current experience and future expectations of mobility and function
  - Preparations in the household for surgery
- **Post-surgery**
  - Experience of aftercare post-surgery
  - Experience of living with SPHERE technology
  - Ask about the adequacy of information received about SPHERE technology
  - Explore how initial expectations of living with the SPHERE technology compared to the experience

Pre-surgery interviews began with an introduction to the aims of the interview, and a discussion of their route to referral, views about the SPHERE sensor system, household constitution and health technology usage. Post-surgery interviews explored care after surgery and living with the SPHERE sensor system. Interviews were audio recorded, transcribed and anonymised.

Using thematic analysis [7], the researcher read and re-read the data to ensure familiarity, coding inductively before sorting coded data into themes [26]. Codes were checked for consistency and validation by a second researcher familiar with the topic area and verified by the clinical study team.

Qualitative evidence shows participant B accordingly reports a fairly good recovery, albeit broken sleep expected for that stage of recovery.

Despite an uncomfortable expected first few days in the immediate post-operative period, participants B and C perceive recovery very differently. Participant B describes a fairly good recovery. We illustrate this with direct quotations below:

**Researcher:** Have your expectations from surgery been met since having the surgery?
**Participant B:** Yes, absolutely... I’m where I should be, I think, at two weeks. The balance of better/uncomfortable is about what you’d expect. So yes, I would say so.

Participant C on the other hand reports wounds complications in the days immediately after surgery

**Researcher:** What is different about your life now compared to before surgery?
**Participant C:** Less active... Yeah, not as active... One day we [participant C and wife] were able to walk up to the lamppost... That’s before all these things [wound complications] started to happen... this was before the bleeding... I get up and hits you a kick

PROMs are useful tools to evaluate the success of interventions and changes in health outcomes based on large cohorts, but as we highlight above, PROMs are limited in characterising recovery on an individual level. We suggest that the continuous data that can be captured therefore from sensor data seeks to strengthen the data provided by PROMs.

## Discussion

In general, the results illustrate that SPHERE system information are a useful adjunct to descriptive information provided by the PROMs in characterising recovery on an individual basis. To consider sleep regularity for instance, the variation in patterns of sleep over the three cases can potentially assist a health professional to understand the impact sleep may have on recovery. Another is movement within the home and the amount of time that they spend outside the home, which, although not directly reflected in metrics of patient recovery, do provide useful ‘actionable’ indicators to the health professional of the extent to which the patient has resumed an everyday routine.

**Fig. 10:**
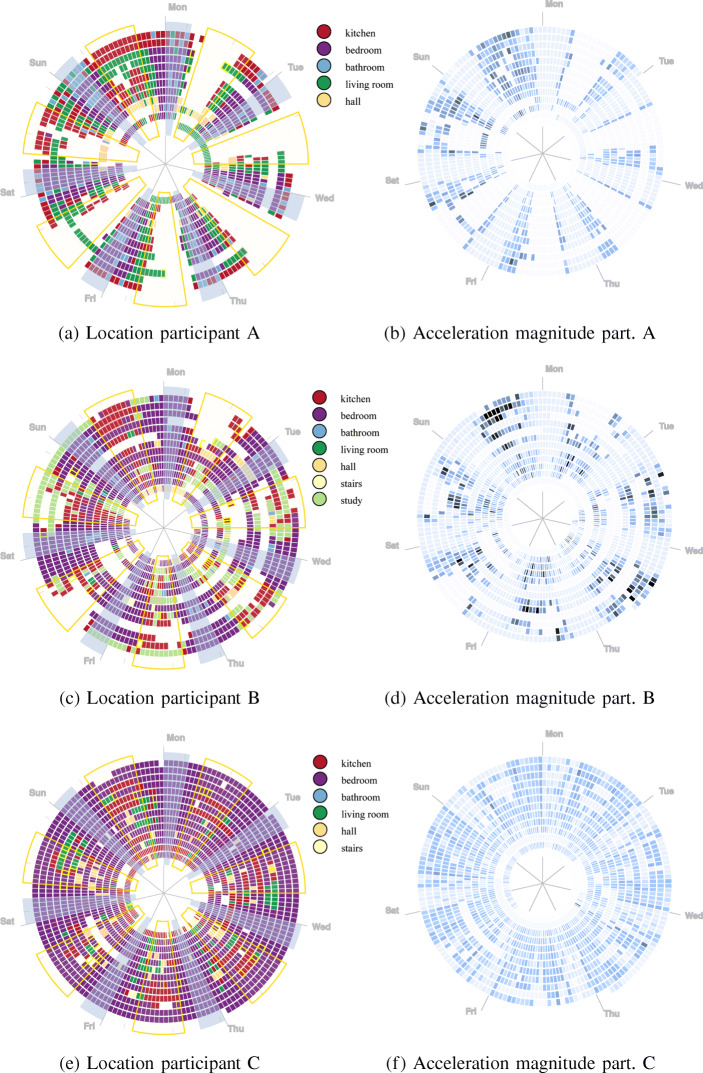
Left: Hourly modal location and dominant activity. Average least active times (L5) and most active (H10) throughout the study are highlighted by grey and yellow segments. Hours during which the dominant component consists of an active activity (walking, stair climbing) are bordered in yellow, whilst standing is bordered in pink. Right: Acceleration magnitude (75th percentile). Spirals are to be read clockwise from inside (start of study) to outside

A significant challenge for the present study is transforming the data obtained from the sensor system to meaningful data for health professionals to infer anything from. Towards this end, we have developed a number of visualisations intended to provide at-a-glance indication of change over a relatively large period of time. The left-hand column of visualisations in Fig. 10 displays participant location within the home, juxtaposing most and least active times, which provides the viewer with a straightforward means to scan for anomalies, such as use of kitchen, study or bathroom during the time of least activity, or lengthy time periods spent in the bedroom during the day. The right-hand column displays acceleration magnitude, filtered to the 75th percentile of standard deviation to facilitate visual review.

## Conclusions and Future Work

The case study has demonstrated that trends in patient movement, posture and location can be reliably and accurately analysed using from RSSI and accelerometer data gathered using the SPHERE sensor network over the time a patient is in recovery from surgery.

RSSI *fingerprints* collected during the technician walk-around activity were sufficient (Table 1) to model distinct locations within the home. The long-term trends in location data provide a picture of diverging patterns of domestic behaviour between patient homes B and C. Whilst B appears to use more locations over time, including time outside the home, patient C is shown to withdraw from many locations in the home, instead spending time in the bedroom.

Routine of activity and passivity is highlighted in movement estimates, with accelerometer magnitude providing the clearest view of true movement levels. Movement data highlights the difference in movement behaviour between patients B (Fig. 7b) and C (Fig. 7c). Whilst B increases movement over time, the patient in home C decreases their overall levels of movement.

Activity classifications, shown in Fig. 9, show distinct patterns of activity for each patient. The results suggest that the method of classification has produced meaningful activity classifications and should provide a basis for an expanded activity set in future work.

Finally, our observations and classifications are partly validated by the self-reported information provided through traditional PROMs and qualitative observations from patient interviews.

The future work of this paper can be divided into three directions: (1) to improve the overall data processing pipeline, (2) to enhance the capability of the system by advanced machine learning and artificial intelligence, (3) to investigate and develop use-cases for systems and data interactions across both patient and clinical stakeholders.

For the overall data processing pipeline, as discussed previously, one of the main issues among the current system is the failure of sensors and hence the loss of certain data. Whilst the direct solution is to detect the failed sensors and fix them during data collection, it might dramatically increase the cost of the system in terms of human resource. Hence, the one of the main areas of future work is, assuming that subsets of certain types of data will be lost during the period of operation of a home-installed sensor network, to develop a processing pipeline that is robust against each known type of missing data.

The training data for location prediction has a uniform prior, as the technicians spend around 50 s on each room that they visit. This means that the models are not biased towards any room in particular. However, this approach may have limitations in that rooms are used to different extents in the real world. In future work, there is a need to investigate alternative location annotation methods, such as near field contact (NFC) tags, for continuous or incremental label collection, which may provide a more realistic distribution of locations. Online training may also help to overcome drift in RSSI data caused by changes to the environment, such as furniture or electronics, which interfere can with blue-tooth signals. Additionally, further parameter tuning for any location classifier may improve upon accuracy reported in this study.

With respect to the machine learning and artificial intelligence techniques, the next step is to further reduce the requirement on the amount of training data, and therefore to build a improved approach to infer the recovery progress of the patients. At the current moment, the recovery of the patient is monitored with extensive reference to the inferred activities and locations, for which a set of well-annotated ground truth data is required to train the classifiers. For this purpose, means of self-annotation have been identified that permit participants to electively contribute further ground truth data. These are currently under evaluation within a healthy control population. However, as such training data is generally difficult to obtain on a large scale, it would be more valuable to further consider approaches that are more efficient on the current available data, and ideally to directly infer the recovery progress without a exact inference over activity and locations.

Finally, as the aim of the system is to provide certain information back to the clinician, and hence to improve the recovery of the patients, we are working to identify potential methods that allow fruitful communication and interaction between the clinician and the patients on the basis of SPHERE data. The visualisation methods displayed in this paper are under evaluation with clinicians through a series of workshops, and we expect to evaluate further approaches to communication of key data to clinicians and to the patient.
